# Morphometrical Study of the Lumbar Segment of the Internal Vertebral Venous Plexus in Dogs: A Contrast CT-Based Study

**DOI:** 10.3390/ani11061502

**Published:** 2021-05-22

**Authors:** Valeria Ariete, Natalia Barnert, Marcelo Gómez, Marcelo Mieres, Bárbara Pérez, Juan Claudio Gutierrez

**Affiliations:** 1 Institute of Pharmacology and Morphophysiology, Austral University of Chile, Valdivia 5090000, Chile; valeria.ariete@gmail.com (V.A.); maildeatty@gmail.com (N.B.); barbaraperez@uach.cl (B.P.); 2 Institute of Veterinary Clinical Sciences, Austral University of Chile, Valdivia 5090000, Chile; mmieres@uach.cl; 3 Department of Anatomy, Physiology and Cell Biology, School of Veterinary Medicine, University of California, Davis, CA 95616, USA; jcgutier@ucdavis.edu

**Keywords:** internal vertebral venous plexus, computed tomography, canine

## Abstract

**Simple Summary:**

The internal vertebral venous plexus (IVVP) is a valveless venous network running inside the vertebral canal. The objective of this study was to morphometrically describe the IVVP, dural sac, epidural space and vertebral canal of the lumbar segment in dogs with enhanced computerized tomography. Six clinically healthy adult dogs were used for the study. Dorsal reconstructed computed tomography (CT) images showed a continuous rhomboidal morphological pattern for the IVVP. The dural sac was observed as an isodense structure with a rounded shape throughout the vertebral canal. The average percentage area occupied by the IVVP between L1 and L7 vertebrae ranged between 6.3% and 8.9% of the area of the vertebral canal, and the dural sac ranged between 13.8% and 72.2% of the vertebral canal. The epidural space accounted between 27.08% and 86.2% of the lumbar vertebral canal. CT venography is a safe technique that allows adequate visualization and evaluation of the lumbar IVVP and adjacent structures in dogs.

**Abstract:**

The internal vertebral venous plexus (IVVP) is a thin-walled, valveless venous network that is located inside the vertebral canal, communicating with the cerebral venous sinuses. The objective of this study was to perform a morphometric analysis of the IVVP, dural sac, epidural space and vertebral canal between the L1 and L7 vertebrae with contrast-enhanced computed tomography (CT). Six clinically healthy adult dogs weighing between 12 kg to 28 kg were used in the study. The CT venographic protocol consisted of a manual injection of 880 mgI/kg of contrast agent (587 mgI/kg in a bolus and 293 mgI/mL by continuous infusion). In all CT images, the dimensions of the IVVP, dural sac, and vertebral canal were collected. Dorsal reconstruction CT images showed a continuous rhomboidal morphological pattern for the IVVP. The dural sac was observed as a rounded isodense structure throughout the vertebral canal. The average area of the IVVP ranged from 0.61 to 0.74 mm^2^ between L1 and L7 vertebrae (6.3–8.9% of the vertebral canal), and the area of the dural sac was between 1.22 and 7.42 mm^2^ (13.8–72.2% of the vertebral canal). The area of the epidural space between L1 and L7 ranged from 2.85 to 7.78 mm^2^ (27.8–86.2% of the vertebral canal). This CT venography protocol is a safe method that allows adequate visualization and morphometric evaluation of the IVVP and adjacent structures.

## 1. Introduction

**Figure 1:**
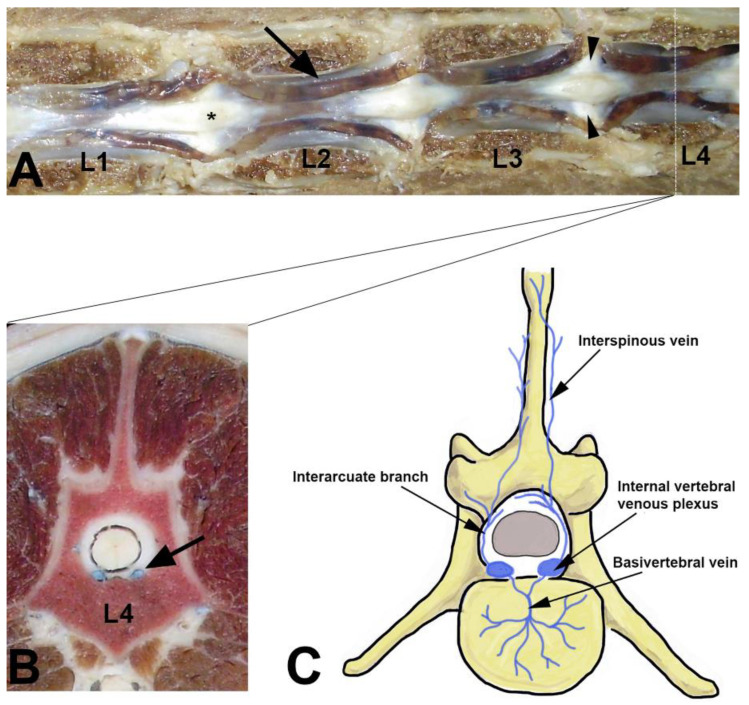
Lumbar internal vertebral venous plexus (IVVP) in a dog. (**A**) Dorsal anatomic view in which the vertebral arch and spinal cord was removed between L1 and L4. The IVVP is observed over vertebral bodies (arrow), intervertebral disk (arrow heads), and dorsolateral to the dorsal longitudinal ligament (asterix), with a typical rhomboidal appearance (arrow). (**B**) Transverse anatomic view of fourth lumbar vertebra where the IVVP (arrow) is located ventrally in the spinal epidural space and dorsal to the L4 vertebral body. (**C**) Schematic representation of some components of the vertebral venous plexus in the dog (Photographs correspond to previous dissections made by the author that do not correspond to the animals examined in this study).

The vertebral venous plexus (VVP) is a thin-walled, valveless venous network that surrounds the entire length of the vertebral column, terminating at the cephalad end in the cerebral venous sinuses [1,2]. According to its position inside or outside of the vertebral canal, the vertebral venous plexus can be divided into three intercommunicating divisions: the internal vertebral venous plexus, external vertebral venous plexus, and basivertebral veins [1]. The internal vertebral venous plexus (*plexus vertebralis internus ventralis*) (IVVP) is also known as the longitudinal venous sinus, vertebral venous sinuses, epidural venous plexus, paravertebral veins, and meningorachidean plexus [3]. The IVVP lies within the vertebral canal, inside the epidural space, and along the dorsal surface of the vertebral bodies and intervertebral disks [1,2] (Figure 1). Cranially, the IVVP communicates with the basilar sinuses at the level of the foramen magnum, and caudally, it extends to the fourth or sixth caudal vertebra [1]. The VVP drains blood from the vertebral column, the paravertebral musculature, the spinal cord, the meninges, and the nerve roots of the spinal nerves [3]. In addition to its normal drainage function, this vascular network can also be a collateral pathway for blood return towards the heart in cases of occlusion or ligation of the caval venous system [4]. Clinically, in humans and animals, rupture of the IVVP has been associated with the etiology of spontaneous spinal epidural hematomas [5,6]. Doberman pinschers with a deficiency of von Willebrand factor (VIII-related antigen) can develop significant spinal epidural hemorrhage due to laceration of the IVVP, which results in progressive neurological deficits [7]. Following Hansen type I intervertebral disk herniation, the extruded nucleus pulposus can be expelled laterally and rupture the IVVP. This can result in bleeding, hematoma formation, and subsequent extradural spinal cord compression [8]. The continuity of the valveless IVVP throughout the length of the neuroaxis enables bacteria and tumor cells to travel from the thorax, abdomen, and pelvis to the head and vertebral column when intrathoracic or intra-abdominal pressure is increased [9]. This phenomenon has been termed paradoxical embolism, retrograde venous invasion, and Batson’s phenomenon [9]. In dogs, metastasis of osteosarcomas and pheochromocytomas into the central nervous system is hypothesized to occur by retrograde venous spread through the VVP. Diskospondylitis, vertebral osteomyelitis, and infectious diskitis in dogs with primary sites of infection elsewhere in the body have been explained by this mechanism [10]. The IVVP may participate in the etiology of fibrocartilaginous embolism of the spinal cord vasculature (also known as embolic myelopathy) in dogs [11]. In humans, the IVVP also participates in the pathophysiology of other spinal cord vascular lesions, such as arteriovenous fistulas and venous malformations [12]. Recently, venous aneurism of the IVVP in a Scottish Deerhound was reported concurrently with severe dilatation of the venous sinuses [13]. Additionally, case reports in dogs have described abnormal enlargement of the IVVP in sighthounds by magnetic resonance imaging (MRI) with clinical signs of radiculopathies and myelopathy [14].

Understanding anatomical vertebral morphometry is an important factor when interpreting the pathological changes associated with several structural spinal diseases. The aims of the present study were to provide an in vivo morphometric description of the normal lumbar IVVP and surrounding vertebral structures using contrast-enhanced computed tomography (CT) in dogs.

## 2. Materials and Methods

### 2.1. Biological Material

For the study, 6 healthy adult dogs were used. The age range was between 2 and 6 years old, and the weight range between 12 and 28 kg, with an average weight of 19.5 kg. The dog breed included 1 Boxer, and 5 mixed-breed, with 3 males and 3 females. Owners provided written consent for research purposes via an authorization form. The experimental study was carried out according to the protocols proposed by the Bioethics Committee of the Austral University of Chile and all procedures were monitored throughout the study by a veterinarian

### 2.2. Anaesthesia Protocol

All CT studies were performed under general anesthesia with the patient positioned in dorsal recumbency. For the study, dogs were food-deprived for 12 h prior to the CT scan. Each dog was premedicated with 0.5 mg/kg of intramuscular xylazine (2% Xylazine, Xylavet 2% Lab. Alfasan, Worden, Holland). Then, animals were cannulated with a 20 G cannula in the cephalic vein to administer a contrast medium in a continuous infusion of saline (0.9% NaCl) in a volume of 10 mg/kg/h. Anesthesia was induced by intravenous administration of propofol (Propofol 1%, PropoFlo; Zoetis, NJ, USA) at a dose of 4 μg/kg and maintained with 2% isoflurane in oxygen.

### 2.3. CT Venography Protocol

For the study, a four-generation CT scanner (Picker PQ 6000, Picker International, Cleveland, OH, USA) was used. For imaging, dogs were positioned in dorsal recumbency with flexed hindlimbs over the CT table. Contiguous CT slices were obtained before and after the application of a nonionic iodinated contrast medium (Iohexol, Omnipaque^®^ 300 mg of iodine/mL, Nycomed Inc, Princeton, NJ, USA) in a total dose of 880 mg/kg IV, applying 587 mg I/kg by bolus, and flow rate of 4 mL/s before the scan. Infusion of an additional 293 mg I/kg as continuous infusion during the scan was administered by means of a constant-rate infusion pump with a flow rate of 0.1 mL/min. Technical parameters included: Slice thickness of 2 mm and an interval of 2 mm and a scan field of view of 180 cm. Settings of the CT scanner included standard resolution, 100 mA and 130 kV per slice. The study area included from L1 to L7 vertebrae. The CT gantry was tilted such that the sections were parallel to intervertebral spaces. All dogs recovered routinely from anesthesia and were clinically normal at 24 and 48 h post CT examination.

### 2.4. Morphometric Analysis

**Figure 2:**
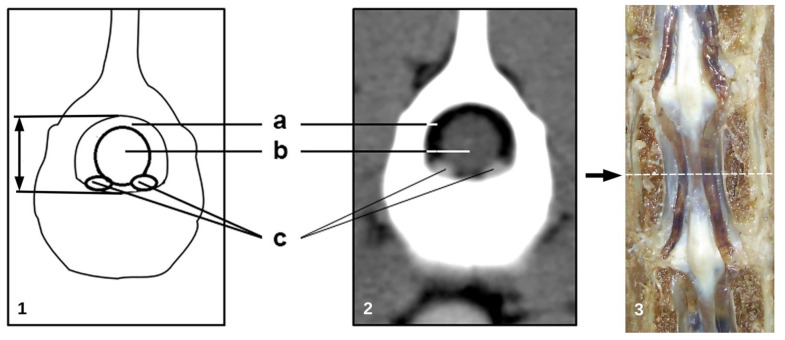
(**1**) Illustration and (**2**) transverse post-contrast CT image at (**3**) the level of L2 vertebra in an adult dog illustrating the measured anatomical parameters. The broken line in the photograph indicates the level of the transverse section where the measurements were performed in the L1-L7 vertebral segment. (**a**) Epidural space, (**b**) dural sac, (**c**) internal vertebral venous plexus.

The CT images were transferred to a computer for morphometric assessment. Measurements were obtained from CT transverse images using standardized soft tissue window settings (window width WW: 250–450; window level WL: 30–50). All measurements were performed between L1 to L7 at mid-vertebral body level. This segment was chosen due to the scarce morphometric information of components of the vertebral canal at this level in dogs. All image data were imported and recorded into the Osirix^®^ DICOM viewer (Pixmeo Inc., Version 3.9.4., 32 Bit, Bernex, Switzerland), a software for morphometric analysis. Anatomical parameters were measured at the mid-level of the vertebral body of the lumbar segment (L1–L7). Measurements were taken in millimeters in the axial planes and were collected three times by the same observer. The following anatomical parameters were determined considering the anatomical structures shown in Figure 2:

− cross sectional area of the vertebral canal (mm^2^);
− cross sectional area of the dural sac (mm^2^);
− total area of the IVVP (area of the right and left IVVP component) (mm^2^);
− area of the epidural space: vertebral canal area minus the area occupied by the dural sac (mm^2^);
− percentage of the IVVP occupying the vertebral canal;
− percentage of the dural sac occupying the vertebral canal;
− percentage of the IVVP within the epidural space;

### 2.5. Statistical Analysis

Data are presented as the mean ± standard deviation and were analyzed using STATGRAPHICS (Centurion XVI version 16.1.12, 32 bits, StatPoint Inc., Rockville, MD, USA) software.

## 3. Results

In all six dogs, appropriate opacification of the vertebral venous component was achieved in all of them. Nonselective CT venography developed in this study allowed adequate visualization and measurement of the IVVP and associated vertebral structures. The contrast injection protocol based on nonionic iodinated contrast medium in a total dose of 880 mg I/kg, 2/3 of which was delivered as an initial bolus before CT image acquisition and 1/3 of which was delivered in the form of a constant-rate infusion pump, resulted in an IVVP average density of 215 UH. Image acquisition time lasted between 25 and 35 min.

### 3.1. Internal Vertebral Venous Plexus

**Figure 3:**
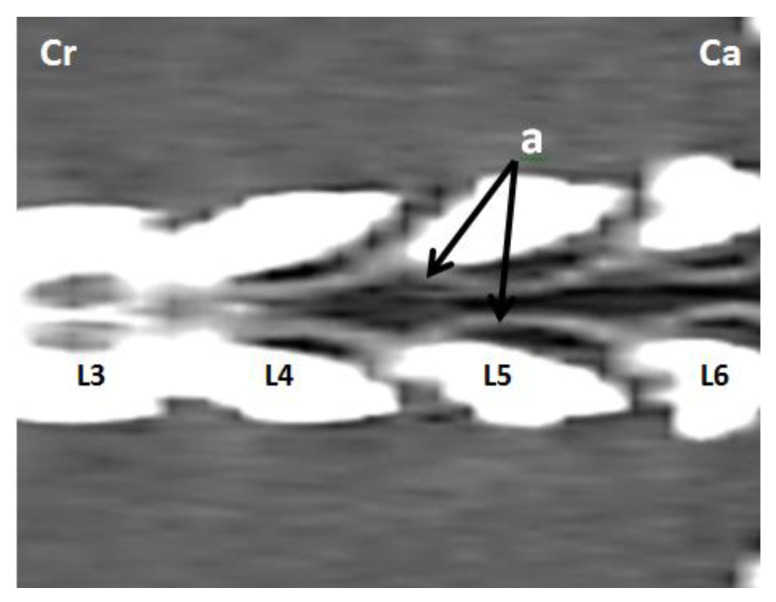
Dorsal reconstruction post-contrast CT images at the L3–L6 lumbar level in an adult medium size dog. (**a**) Intervertebral venous plexus, (**cr**) cranial, (**ca**) caudal.

**Figure 4:**
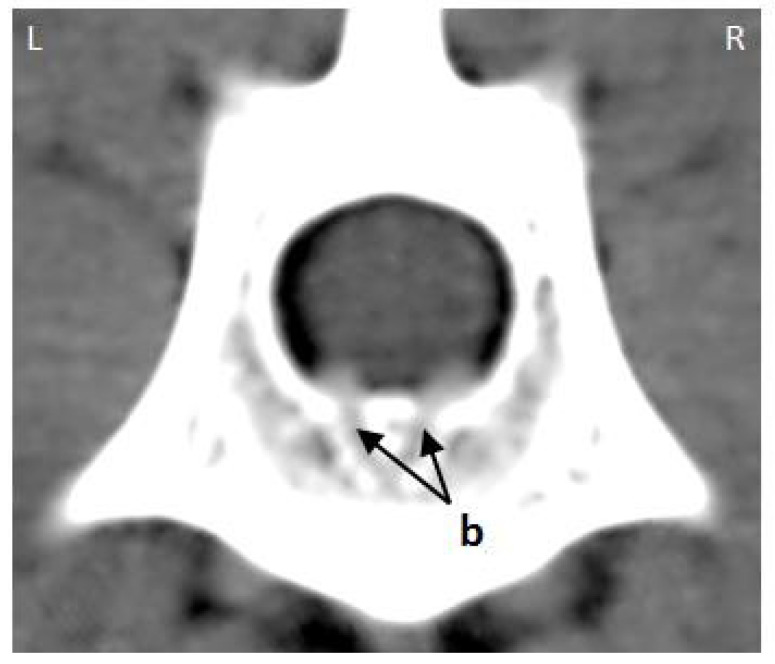
Transverse post-contrast CT image of the mid-portion of the L3 vertebral body from an adult medium size dog. (**b**) basivertebral veins, (**L**) left, (**R**) right.

**Figure 5:**
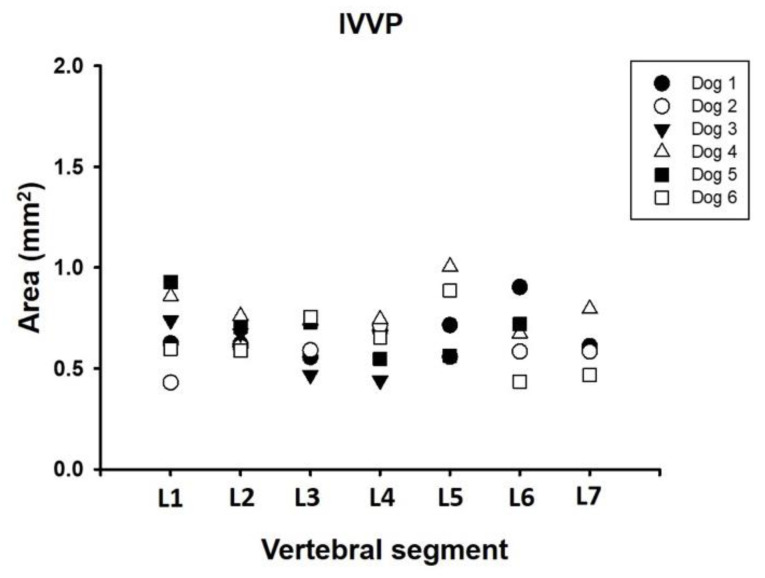
Area dimension of the internal vertebral venous plexus (IVVP) between L1 and L7 vertebral segment in six adult medium size dogs.

The internal vertebral venous plexus (IVVP) was observed as two symmetric longitudinal hyperdense channels, oval in shape, located in the epidural space, dorsal to the vertebral bodies, and ventral to the dural sac (Figure 2). In dorsal reconstruction CT images, it was possible to appreciate their rhomboidal distribution within the vertebral canal (Figure 3). Additionally, CT transverse images obtained at the level of the middle portion of the vertebral body allowed visualization of the basivertebral veins and tributaries of the IVVP (Figure 4). The IVVP between the L1 and L7 vertebrae had an average area of 0.66 mm^2^, with a minimum value recorded of 0.43 mm^2,^ and the maximum of 1.01 mm^2^. In vertebra L7, the IVVP presented the lowest average with a 0.61 mm^2^ (±0.1) area, and L5 vertebrae presented the highest IVVP average area with a 0.74 mm^2^ (±0.2) area (Table 1, Figure 5). The IVVP occupies an average of 7.67% of the L1–L7 vertebral canal. In the L1 vertebra, there was the highest percentage of the IVVP in relation to the vertebral canal, with 8.9%, while in the L4 vertebra, IVVP occupied the lower percentage, with 6.3% of the vertebral canal (Table 2).

### 3.2. Vertebral Canal

**Figure 6:**
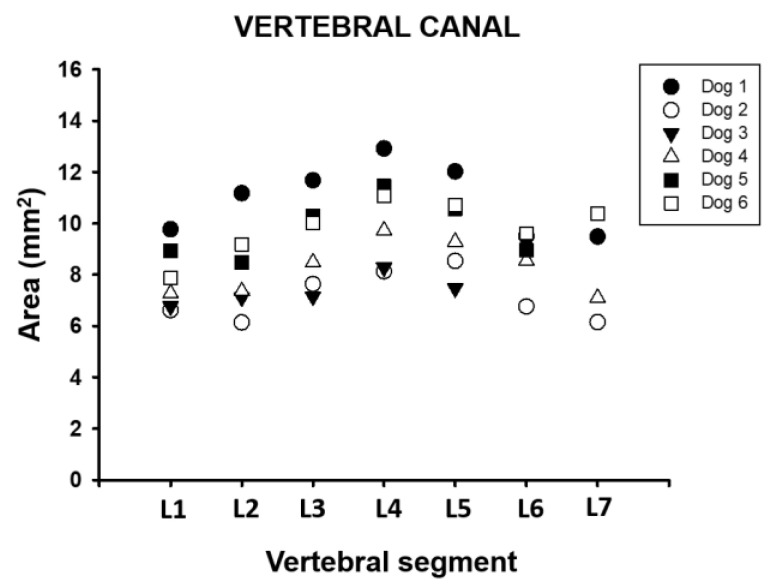
Area dimension of the vertebral canal between L1 and L7 vertebral segment in six adult medium size dogs.

The vertebral canal measured an average of 8.94 mm^2^ (±1.7) between the L1 and L7 vertebral segments, the minimum value recorded was 6.14 mm^2,^ and the maximum was 12.92 mm^2^. Vertebra L1 had the lowest recorded value with a 7.87 mm^2^ (±1.3) area, while the L4 vertebra had the highest value with a 10.27 mm^2^ (±1.9) area (Table 1, Figure 6).

### 3.3. Dural Sac

**Figure 7:**
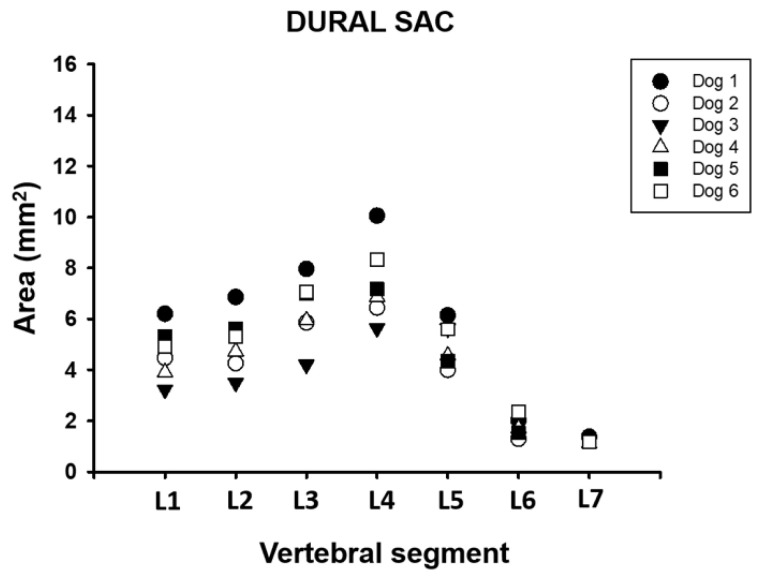
Area dimension of the dural sac between L1 and L7 vertebral segment in six adult medium size dogs.

The dural sac had an average area of 4.83 mm^2^ (±2.1) between vertebral segments L1 and L7. The minimum recorded value was 1.11 mm^2,^ and the maximum value was 10.05 mm^2^. The dural sac in the L7 vertebra had the lowest value with a 1.22 mm^2^ (±0.1) area, and the L4 vertebra had the highest average with a 7.42 mm^2^ (±1.6) area (Table 1, Figure 7). The dural sac occupied between 13.8% and 72.2% of the vertebral canal between the L1 and L7 vertebral segments in these dogs (Table 2). It is also possible to appreciate that there is an increase in the occupancy rate toward L4, where it reaches its maximum value, and then it decreases toward L7, where the lowest average value is recorded.

### 3.4. Epidural Space

**Figure 8:**
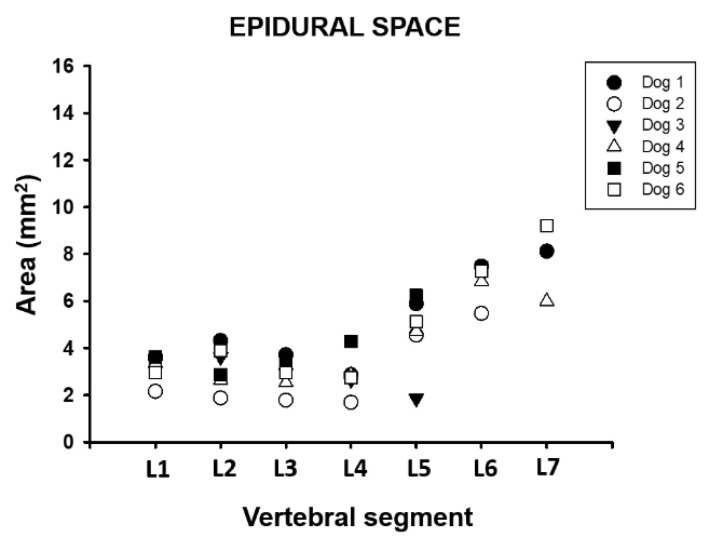
Area dimension of the epidural space between L1 and L7 vertebral segment in six adult medium size dogs.

The average area of the epidural space between segments L1 and L7 ranged from 2.85 (±0.8) to 7.78 (±1.6) mm^2^, corresponding to 27.8% to 86.2% of the vertebral canal (Table 1 and Table 2, Figure 8).

## 4. Discussion

The nonselective CT venography technique developed in this study allowed adequate visualization and measurement of the lumbar IVVP and associated vertebral structures in a group of dogs. The contrast injection protocol based on nonionic iodinated contrast medium in a total dose of 880 mg I/kg, 2/3 of which was delivered as an initial bolus before CT image acquisition and 1/3 of which was delivered in the form of constant-rate infusion during CT acquisition, resulted in an IVVP density of 215 UH. According to different studies, for optimal vasculature viewing in CT examinations, it is necessary to use a window level ranging between 200 and 400 UH and a window width ranging between 200 and 2000 UH, depending on the vascular concentration of contrast medium [15]. There are various doses and administration protocols for contrast administration published for CT venography in dogs. There have been protocols with values ranging from 720 mg I/kg to 814 mg I/kg in different studies, including the study of IVVP cervical level, diagnosis of portosystemic shunts, multiple vena cava anomalies, pancreatic TC analysis, and evaluation of pulmonary embolisms [16,17,18,19,20,21]. Although high doses of contrast medium were used in this CT venographic study, no adverse effects were observed in all animals examined. However, it is necessary to consider that large doses of contrast medium in angiographic studies may be associated with major risk of contrast agent hypersensitivity and renal insufficiency [22].

The IVVP appearance on the CT images consisted of two symmetrical oval hyperatenuated structures with defined margins located on the floor of the vertebral canal (Figure 2). The description observed in this study evidenced a rhomboidal distribution with a metameric organization of the venous system consistent with previous publications in dogs [1,17,18]. Regarding morphometry of the IVVP, the average area measurements for the lumbar segment (from 0.61 to 0.74 mm^2^) were lower than values published previously for the cervical segment in dogs, where an average area of 1.33 mm^2^ was recorded in similarly-sized dogs [18]. In addition, the progressive cranio-caudal decrease in the diameter of the IVVP described previously was also observed in previous studies [3,18]. The lumbar IVVP constituted, on average, 7.67% of the area of the vertebral canal and 19.69% of the epidural space, lower values than those reported for the cervical segment values, where these vessels occupy 12.4% of the vertebral canal and 30.61% of the epidural space [18]. These venous trunks tend to decrease in size gradually from vertebrae T4 to L7, away from each other at intervertebral spaces and converging in the center of the vertebral bodies. The size of the IVVP in the L5–L7 segment was smaller and variable, therefore values of the structures in this area should be taken with caution.

In humans, the IVVP is smaller in the cervical area, being bulkier and pronounced as it descends through the vertebral canal, reaching a maximum size at the level of the L4–L5 vertebrae [5]. This difference is most likely related to the postural difference between humans and canids [5]. Dissection studies in humans determined that the anterior IVVP, equivalent to the ventral IVVP in dogs, has a mean length of 103 mm (range 43–153 mm) and a maximum width of 5.8 mm. The maximum caliber of the anterior IVVP in humans is at the thoracolumbar segments (mean maximum width 7.2 ± 2.0 mm) [22].

Previous anatomical descriptions of the IVVP have been performed for humans, dogs, domestic cats, rodents, and even reptiles using various imaging modalities ranging from conventional radiography to more modern techniques, such as magnetic resonance imaging (MRI) and CT [17,18,23,24,25,26]. In vivo imaging studies are more appropriate to analyze the vascular anatomy in animals because of vein collapse in cadaver specimens.

In humans, epidural venous engorgement in the lumbar spine, including epidural varices, may cause pain in the lower back and radiculopathy secondary to nerve root impingement [27]. The diagnosis of IVVP engorgement is often mistaken for a herniated disc on radiologic interpretation, and the true diagnosis is finally made in the surgical intervention. In dogs, vertebral venous system abnormalities have been identified on MRI in 12% of all sighthounds, but the underlying cause is unknown [14]. Recently, severe dilation of the right IVVP causing significant compression of the spinal cord and nerve roots was identified in an adult Scottish Deerhound [13]. Bilateral vertebral venous sinus thrombosis and dilation causing cervical spinal cord compression were also recently detected in a 10-year-old male mixed dog [28]. In another study, the visibility of the IVVP was significantly (*p* < 0.001) different between Great Danes with and without clinical signs of cervical spondylomyelopathy [29]. Variation in the size of the IVVP in the central nervous system (CNS) can be explained by the Monro-Kellie doctrine, which establishes an inverse relationship between cerebrospinal fluid (CSF) volume and intracranial blood volume [30]. Hence, as CSF is removed from the intracranial compartment, more blood enters the intracranial compartment. Because the intracranial compartment and vertebral canal are nearly a closed system, the principles of the Monro-Kellie doctrine can be extended to the vertebral canal. As expected, the size of the dural sac increased with increased intracranial CSF or blood volume and decreased with decreased intracranial CSF or blood volume [30]. In the brain, the dura mater is closely opposed by bone, whereas in the vertebral canal, the dura mater has the IVVP and fat separating it from the fixed bony vertebral canal. As the epidural fat is constant and likely has little movement in and out of the neural foramen, the IVVP is most likely to change in size with changes in dural sac volume [30].

In relation to the dural sac, the increase in the average area recorded at the L4 vertebra level is associated with medullary expansion of the L4-S3 segment, known as lumbar enlargement (*intumescentia lumbalis*) [1]. From this lumbar enlargement, the nervous roots of the lumbosacral plexus emerge, which can be seen in the transverse images from the L5 vertebra caudal. The dural sac area values recorded for each vertebral segment, with the exception of L4, are smaller than those published by Gómez et al. (2005) for the cervical segment in dogs, where an average area of 6.40 + 1.0 mm^2^ was recorded. A CT study of the dural sac in humans indicated that between the L4 and L5 segments, the average area of the dural sac was >100 mm^2^, which was higher than that recorded in the present study for the same vertebral segment in dogs [31]. In humans, a dural sac cross-sectional area < 100 mm^2^ and dural sac anteroposterior diameter <10 mm are frequently considered to assess the severity of spinal canal stenosis [31,32]. The percentage of the cross-sectional area of the dural sac found in our study was between 50 and 72% of the vertebral canal between L1 and L4. These values were similar to those reported by Lim et al. (2018) [33], with values of 64% in a group of 12 normal Beagle dogs. In this study, a significant reduction in the ratio cross-sectional area of the dural sac (40%) in patients with different spinal disorders (IVDD, spinal tumors, hematomas, etc.) to that of the vertebral canal was observed [33].

The vertebral canal area measured 8.94 mm^2^ between the L1 and L7 vertebral segments on average. Similar results were observed in a morphometric CT study of the thoracic spine performed in 13 German Shepherd dogs for the T2–T13 vertebral level (8.36 ± 4.03 mm^2^) [34]. A decrease in vertebral canal diameter and/or area that results in compression of spinal cord and/or nerve roots is termed absolute stenosis, whereas a diameter that is less than normal but does not cause compression of neural elements is termed relative stenosis [35,36]. Relative vertebral canal stenosis results in decreased available space for the spinal cord to compensate for extradural space-occupying conditions. Relative vertebral canal stenosis therefore predisposes animals to develop clinical signs when relatively mild space-occupying pathologies, such as age-related intervertebral disc protrusion or ligamentous hypertrophy, occur [35,36].

The percentage of the vertebral canal of the spinal epidural space was minimal between L1 and L4 (27–41%), indicating that these segments are more subject to clinically significant epidural compressive lesions and that L5–L7 had a greater amount of vertebral canal available for the dural sac (47–87%). The large size of the epidural space between L5 and L7 also provides a secure space for epidural instruments or devices (i.e., catheters) in this region. The spinal epidural space surrounds the dural sac and is limited dorsally by the epidural fat, ligamentum flavum and periosteum, ventrally by the dorsal longitudinal ligament, IVVP and vertebral bodies, and laterally bordered by the vertebral pedicles and intervertebral foramina [1]. In MRI and CT imaging studies, severe stenosis is usually associated with subjective signs of the absence of epidural fat [37,38,39]. There are no quantitative studies using CT images that evaluated the dimensions or proportion of the lumbar epidural space in dogs.

Limitations of the present study were associated with the small number of dogs included and the variety of dogs of different sizes evaluated. Further studies to increase the study population and standardize various breeds to allow analysis by breed (small, medium, large, and giant) size are necessary. These data should thus be interpreted with caution given that measurements were only acquired at the mid-vertebral level and did not include the intervertebral cross-sectional area parameters.

## 5. Conclusions

This preliminary study provides reference values of the lumbar IVVP and adjacent structures in a group of dogs. The IVVP constitutes a complex vascular network of the vertebral column that has recently received attention in veterinary medicine. The understanding of its anatomy and morphometry is necessary to adequately diagnose new pathological entities that involve this venous plexus and in preoperative imaging evaluation of the venous morphometry may be useful to avoid complications related with vascular structures.

## Figures and Tables

**Table 1:** Average area (mm^2^) and standard deviation of the IVVP, vertebral canal, and dural sac and epidural space between the L1 and L7 vertebral segments in six adult medium size dogs.

Morphometric Measures	Vertebral Segment						
	L1	L2	L3	L4	L5	L6	L7
Vertebral canal	7.87 ± 1.3	8.24 ± 1.8	9.21 ± 1.7	10.27 ± 1.9	9.77 ± 1.7	8.68 ± 1.2	8.28 ± 2
Dural sac	4.67 ± 1.1	5.04 ± 1.2	6.34 ± 1.3	7.42 ± 1.6	5.04 ± 0.9	1.78 ± 0.4	1.22 ± 0.1
IVVP	0.7 ± 0.2	0.66 ± 0.1	0.64 ± 0.1	0.63 ± 0.1	0.74 ± 0.2	0.66 ± 0.2	0.61 ± 0.1
Epidural space	3.2 ± 0.6	3.2 ± 0.9	2.87 ± 0.7	2.85 ± 0.8	4.72 ± 1.6	6.9 ± 0.8	7.78 ± 1.6

**Table 2:** Average percentage (%), minimum and maximum of the vertebral canal corresponding to the dural sac, internal vertebral venous plexus (IVVP), and epidural space between vertebrae L1 and L7 in six adult dogs.

Morphometric Measures	Vertebral Segment						
	L1	L2	L3	L4	L5	L6	L7
Dural sac	59 (47.7–67,4)	61.3 (49.1–69.4)	68.8 (58.8–76.6)	72.2 (62.6–77.8)	52.6 (46.8–75.3)	20.4 (17–21.2)	13.8 (11.3–15.7)
IVVP	8.9 (6.4–11.8)	8.3 (5.5–10.3)	7.1 (4.8–8.7)	6.3 (4.8–8.8)	7.4 (5.3–10.8)	7.7 (4.5–9.5)	7.9 (4.5–11.2)
Epidural space	41 (32.6–52.3)	38.7 (30.6–50.9)	31.2 (23.4–41.2)	27.8 (20.8–37.4)	47.4 (24.7–58.9)	79.6 (75.6–83)	86.2 (84.3–88.7)
